# Phenotypic and molecular characterisation of a novel species, *Mycobacterium hubeiense* sp., isolated from the sputum of a patient with secondary tuberculosis in Hubei of China

**DOI:** 10.1017/S0950268820000436

**Published:** 2020-02-14

**Authors:** Xiaoli Yu, Hui Zheng, Fang Zhou, Peng Hu, Hualin Wang, Na Li, Juncai He, Peidong Wang, Lu Zhang, Hongsheng Men, Jie Xiang, Shulin Zhang

**Affiliations:** 1 School of Biology and Pharmaceutical Engineering, Wuhan Polytechnic University, Wuhan, China; 2 Department of Immunology and Microbiology, Shanghai Jiao Tong University School of Medicine, Shanghai, China; 3 Wuhan Medical Treatment Center, Wuhan, China; 4 Department of Veterinary Pathobiology, University of Missouri, Columbia, Missouri, USA; 5 Tuberculosis Research Center, Shanghai Public Health Clinical Center, Shanghai, China

**Keywords:** 16S rRNA, genomic analysis, nontuberculous mycobacteria, phenotype, *rpoB*

## Abstract

A new fast-growing mycobacterium, designated strain QGD101^T^, was isolated from the sputum of an 84-year-old man suspected of tuberculosis in Wuhan Medical Treatment Center, Hubei, China. This strain was a gram-staining-negative, aerobic, non-spore-forming and catalase-positive bacterium, which was further identified as the NTM by PNB and TCH tests. The moxifloxacin and levofloxacin exhibited strong suppressing function against QGD101^T^ with MIC values of 0.06 and 0.125 µg/ml after drug susceptibility testing of six main antimicrobial agents on mycobacteria. Based on the sequence analysis of 16S rRNA, *rpoB*, *hsp65* and 16S-23S rRNA internal transcribed spacer, the strain QGD101^T^ could not be identified to a species level. *Mycobacterium moriokaense* ATCC43059^T^ that shared the highest 16S rRNA gene sequence similarity (98%) with strain QGD101^T^ was actually different in genomes average nucleotide identity (78.74%). In addition, the major cellular fatty acids of QGD101^T^ were determined as C18:1*ω*9c, C16:0 and C18:2*ω*6c. The DNA G + C content was 64.9% measured by high performance liquid chromatography. Therefore, the phenotypic and genotypic characterisation of this strain led us to the conclusion that it represents a novel species of mycobacteria, for which the name *Mycobacterium hubeiense* sp. nov. (type strain QGD101^T^ = CCTCCAA 2017003^T^ = KCTC39927^T^) was proposed. Thus, the results of this study are very significant for the clinical diagnosis of tuberculosis and future personalised medicine.

## Introduction

Non-tuberculous mycobacteria (NTM) are a large family of acid-fast bacteria, which can be opportunistic pathogens and cause pulmonary disease resembling tuberculosis, lymphadenitis, skin disease or disseminated disease [1–4]. Until now, more than 160 NTM species have been found in List of Prokaryotic names with Standing in Nomenclature (http://www.bacterio.net/mycobacterium.html). Members of NTM are able to be distinguished from photochromogens, scotochromogens and nonchromogens. NTM can also be classified based on the rate of growth [5–7]. NTM are widely distributed in the environment, particularly in wet soil, marshland, streams, rivers and estuaries [8, 9].

In clinical practice, NTM can cause the TB-like clinical presentations. Importantly, most NTM (except *M. kansasii*) are inherently resistant to or only partially susceptible to the standard anti-tubercular drugs. Thus, the diagnosis of NTM infection is critical for choosing effective treatment [10]. In this study, a clinical strain QGD101^T^ was isolated from the sputum sample of a patient suspected of tuberculosis during July 2012 in Wuhan Medical Treatment Center, Hubei, China (30°67′N 114°29′E). By the image findings and medical history, the case was primarily diagnosed as secondary suspected tuberculosis. The patient had the pathological characteristics such as pulmonary fibrosis cavity, hemoptysis, etc., and the commonly used anti-tuberculosis drugs isoniazid and rifampicin had no obvious effect on conventional treatment. The pathogen culture for mycobacteria was positive. By the use of molecular identification and evolutionary analysis, this mycobacterium was identified as a new species. The results of this study have a great significance for clinical diagnosis of emerging diseases and the replenishment of new mycobacterial resources.

## Materials and methods

### Ethics statement

Ethical approval is granted by the Ethics Committee of Wuhan Polytechnic University (ID Number: 20120720006). The sputum sample was obtained from a patient suspected of tuberculosis in Wuhan Medical Treatment Center in Hubei Province, on 20 July 2012.

### Microbiological analysis

The early morning sputum specimens were collected from an 84-year-old man diagnosed as secondary tuberculosis and were processed with the standard protocol [11]. After decontamination, each sample was cultured onto Löwenstein–Jensen (L–J) media at 37 °C for 8 weeks. The cultures were inspected weekly and growth was examined by visual inspection for colonies. Negative slides were then confirmed by Ziehl–Neelsen staining.

### Biochemical and physiological-testing

Biochemical and physiological-testing, including iron uptake, urease testing, nitrate reduction and pyrazinamidase testing [12], were performed for QGD101T, *Mycobacterium barrassiae* CIP 108545T and *Mycobacterium moriokaens*e ATCC 43059T. Catalase activity was determined using bubble production H_2_O_2_ solution [13].. Other biochemical testing was carried out using VITEK 2 ANC and VITEK 2 GP testing kits according to the protocols of the manufacturer (bioMerieux). Growth characteristics at 28 °C, 37 °C and 42 °C were respectively assessed after 5 days of incubation in L–J medium.

### The drug susceptibility testing (DST) for QGD101^T^

According to the resazurin microplate assay [14], the commonly used antimicrobial agents such as rifampin (RIF), ethambutol (EMB), streptomycin, levofloxacin (LVX), kanamycin (KAN), azithromycin (AZI) and moxifloxacin (MOX) were respectively used to determine the MIC value of strain QGD101^T^.

### HPLC analysis

For quantitative analysis of the cellular fatty acid composition, strain QGD101^T^ was cultured at 37 °C for 5 days. The DNA G + C content was determined by means of reversed-phase high performance liquid chromatography, which was according to the protocol described by Mesbah *et al*. [15].

### DNA extraction, PCR amplification and sequencing

Genomic DNA of *M. tuberculosis* isolate QGD101^T^ was extracted by the classical phenol–chloroform method and stored at −20 °C. Oligonucleotide primers were designed using Primer 5.0 (PREMIER Biosoft, Palo Alto, CA, USA) and Oligo 6 software (Molecular Biology Insights, Inc., Cascade, CO, USA). Amplification of the 16S rRNA gene and 16S-23S rRNA internal transcribed spacer (ITS) sequence was performed as previously described [10]. The *hsp65* gene was amplified using primers Tb11 (5′-ACCAACGATGGTGTGTCCAT-3′) and Tb12 (5′-CTTGTCGAACCGCATACCCT-3′) targeting positions 396–836 of the gene sequence of *M. tuberculosis* [16]. The same experimental protocol was used for amplification with the exception that annealing was performed at 57 °C for 1 min. Amplification of *rpoB* was done with primers MycoF (5′-GGCAAGGTCACCCCGAAGGG-3′; base positions 2573 to 2592) and MycoR (5′-AGCGGCTGCTGGGTGATCATC-3′; base positions 3316 to 3337) in two conserved regions flanking the most variable *rpoB* region [17]. Direct sequencing was performed on amplified products with the 3730xl DNA Analyser. Similarity values and percent divergence of the 16S rRNA gene were calculated using MegAlign of Lasergene (DNA-Star, Madison, WI, USA).

### Sequence analysis and phylogenetic classification

Molecular typing and species identification were performed using BLAST search (http://www.ncbi.nlm.nih.gov/BLAST/). Multiple-sequence alignment was performed in MEGA version 7.0.18. Sequences were then trimmed to start and finish at the same nucleotide position for phylogenetic analysis. The sequences of the 16S rRNA, *rpoB*, *hsp65* genes and ITS sequence of the isolated strain were compared with available sequences from GenBank using the BLAST program to determine their approximate phylogenetic affiliation. The 16S rRNA phylogenetic trees based on mycobacterial species were constructed using neighbour-joining algorithm following Kimura 2-parameter correction and maximum likelihood methods in MEGA 7.0.18. The significance of the branching order was estimated by the bootstrap method based on 1000 replications.

### Genomic analysis

The complete genome sequence of strain QGD101^T^ was determined using the Illumina Miseq. The reads were assembled de novo using SOAPdenovo version 2.04 and the gaps of the assembled sequence were closed using GapCloser version 1.12, and the average nucleotide identity (ANI) value was calculated using JSpeciesWS [18].

## Results

### Genotype identification analysis

The strain QGD101^T^ shares 99% identity with *M. barrassiae* CIP108545T and *M. moriokaense* ATCC43059T in the 16S rRNA gene. It shares 96% identity in the *rpoB* gene with *M. barrassiae* CIP108545 and *M. moriokaense* CIP105393. The ITS sequence analysis showed that this strain shares 85% identity with *M. chubuense* NBB4. The *hsp65* gene sequence analysis also showed that this strain shares 92% identity with *M. moriokaense*.

**Fig. 1.**
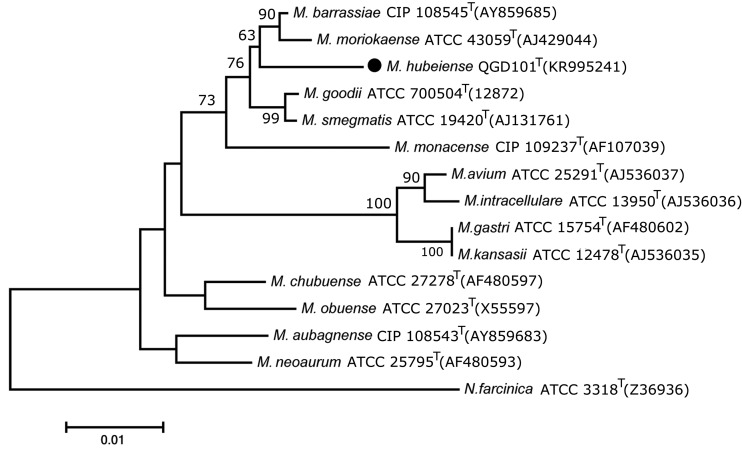
Neighbour-joining trees based on 16S rRNA gene sequences of strain QGD101^T^ and some type strains. The tree was rooted with *N. farcinica* ATCC3318T. Percentages indicated at nodes represent bootstrap levels supported by 1000 resampled datasets; values <50% are not shown. The significance of the branching order was estimated by the bootstrap method calculated 1000 replications. Bar, 0.01 substitutions per nucleotide position.

*Note*: ‘M.’ is the abbreviation of ‘Mycobacterium’, and ‘N.’ is the abbreviation of ‘Nocardia’.

**Fig. 2.**
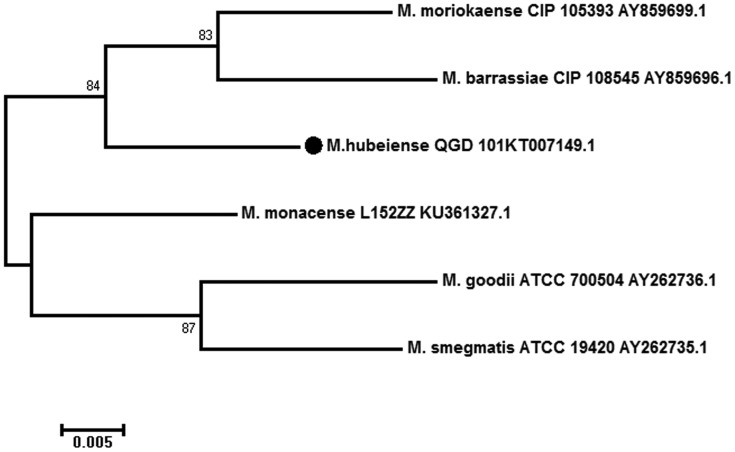
Neighbour-joining trees based on *rpoB* sequences of strain QGD101^T^ and some type strains. Percentages indicated at nodes represent bootstrap levels supported by 1000 resampled datasets; values <50% are not shown. The significance of the branching order was estimated by the bootstrap method calculated 1000 replications. Bar, 0.1 substitutions per nucleotide position.

Results of *rpoB* gene identification are more approximate to the 16S rRNA gene phylogenetic classification. The neighbour-joining tree (Fig. 1) indicated that strain QGD101^T^ was a member of the genus *Mycobacterium* and formed a subcluster with *M. barrassiae* CIP108545T and *M. moriokaense* ATCC43059T with a 63% bootstrap value. Also, the *rpoB* NJ tree showed that QGD101^T^ was closely related to *M. barrassiae* CIP108545T and *M. moriokaense* CIP 105393T, both with an 84% bootstrap value (Fig. 2).

A GenBank accession number for the 16S rRNA, *hsp65* and *rpoB* gene sequence and 16S-23S rRNA intergenic spacer sequence of strain QGD101^T^ are KR995241, KT007146, KT007149 and KR995223, respectively.

### Comparative analysis of genomic sequences

**Fig. 3.**
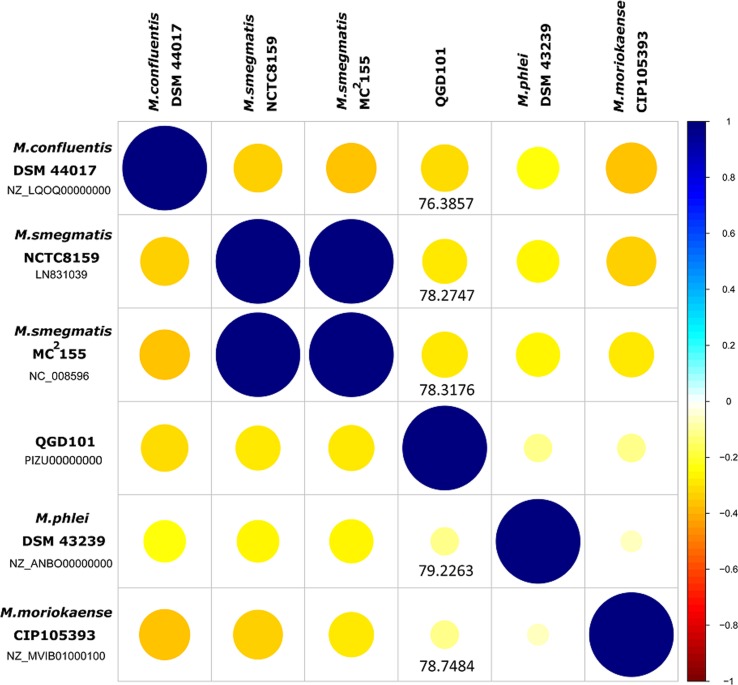
Correlation plot based on QGD101^T^ and reference strains ANI values.

The genomic analysis of QGD101^T^ was obtained by genome sequencing, and the whole sequence of QGD101^T^ has been upload on Genbank. The GC content in the QGD101^T^ gene region is 66.99%, while GC content in the intergenetic region is 62.49%. To evaluate the similarity between genome sequences, ANI values were analysed between the strain and reported genomes of closest species (Fig. 3). The strain QGD101^T^ showed 78.75% ANI values compared to the closest reference strain *M. moriokaense* ATCC43059^T^ (NZ_MVIB01000100). These DNA relatedness and ANI results indicate that strain QGD101^T^ represents a novel genomic species that is distinct from its closest relatives.

This Whole Genome Shotgun project has been deposited at DDBJ/ENA/GenBank under the accession PIZU00000000. The version described in this paper is version PIZU01000000.

### Phenotypic identification analysis

The phenotypic and biochemical characteristics of strain QGD101^T^ are presented in Table 1. The strain QGD101^T^ can grow on media containing either TCH or PNB, indicated that strain QGD101^T^ is a fast-growing NTM.

**Table 1.** VITEK 2 GP and VITEK 2 ANC test kits results

VITEK 2 GP test	*M. hubeiens*e	VITEK 2 ANC test	*M. hubeiense*
D-amygdalin	−	ELLMAN	−
Phosphatidylinositol Phospholipase C	−	Phenylalanine arylamidase	+
D-xylose	−	D-cellobiose	−
Arginine Dihydrolase 1	−	Arbutin	−
*β*-Galactosidase	−	5-Bromo-4-chloro-3-indoxyl-*β*-glucoside	−
*α*-glucosidase	−	5-Bromo-4-chloro-3-indoxyl-*β*-glucuronide	−
Ala-Phe-Pro arylamidase	−	*β*-Galactopyranose indoxyl	−
Cyclodextrin	−	*α*-Arabinosidase	−
L-aspartate arylamidase	−	5-Bromo-4-chloro-3-indoxyl-*α*-galactoside	−
*β*-Galactopyranose	−	*β*-Mannosidase	−
*α*-Mannosidase	−	Arginine GP	−
Phosphatase	−	Pyruvate	(–)
Leucine arylamidase	+	Maltotriose	−
L-proline arylamidase	−	Esculin hydrolyse	−
*β*-Glucuronidase	−	*β*-D-fucosidase	−
*α*-Galactosidase	−	5-Bromo-4-chloro-3-indoxyl-*β*-N-acetyl-glucosamide	−
L-pyrrolidonyl-arylamidase	−	5-Bromo-4-chloro-3-indoxyl-*α*-mannoside	−
*β*-Glucuronidase	−	*α*-L-fucosidase	−
Alanine arylamidase	+	L-arabinose	−
Tyrosine arylamidase	+	d-Ribose 2	−
D-sorbitol	−	Phenylphosphonate	−
Urease	−	*α*-L-arabinofuranosidase	−
Polymixin B resistance	−	GRAM	+
D-galactose	−	MORPH	−
D-ribose	−	AERO	+
L-lactate alkalinisation	−		
Lactose	−		
N-acetyl-D-glucosamine	−		
D-maltose	−		
Bacitracin resistance	−		
Novobiocin resistance	−		
6.5% NaCl	−		
D-mannitol	−		
D-mannose	−		
Methyl-B-D-glucopyranoside	−		
Pullulan	−		
D-raffinose	−		
O/129 resistance	−		
Salicin	−		
Sucrose	−		
D-trehalose	+		
Arginine dihydrolase 2	−		
Optochin resistance	−		

+, positive; −, negative.

The differential characteristics between strain QGD101^T^ and its reference species in the genus *Mycobacterium* are shown in Table 2. The strain QGD101^T^ could also grow on sodium glutamate glucose agar but not on MacConkey agar. *M. barrassiae*, *M. moriokaense* and strain QGD101^T^ were all able to grow at 42 °C, however, none of them could produce pigment. All three strains showed positive activities for urease, 5% NaCl tolerance, iron uptake, nitrate reductase and negative activities for arylsulfatase, akaline phosphatase and esculin. There are also different characteristics among the three species. For example, *M. barrassiae* and *M. moriokaense*, but not strain QGD101^T^, showed positive activities for the pyrrolidonyl arylamidase test. For the *α*-glucosidase test, only *M. moriokaense* showed positive activities. For their carbon source, *M. barrassiae* was able to assimilate both d-sorbitol and l-arabinose except d-mannitol. *M. barrassiae* was able to use l-arabinose only. However, none of the three chemicals could be used by strain QGD101^T^ as its sore carbon source. These differences clearly demonstrated that the strain QGD101^T^ is different from *M. moriokaense* and *M. barrassiae*, other biochemical characteristics of strain QGD101^T^ are shown in Table 2.

**Table 2.** Differential characteristics of strain QGD101^T^ and closely related species

Characteristic	*M. barrassiae*	*M. moriokaense*	Strain QGD101^T^
Growth at 42 °C	+	+	+
Pigmentation	－	－	－
Arylsulfatase	－	－	－
Catalase	+	+	+
Urease	+	+	+
5% NaCl tolerance	+	+	+
Iron uptake	+	+	+
Nitrate reductase	+	+	+
Pyrrolidonyl arylamidase	±	±	－
Alkaline phosphatase	－	－	－
*β*-Galactosidase	－	+	－
*α*-Glucosidase	－	+	－
Esculin	－	－	－
Utilisation of the following as a sole carbon source：			
D-mannitol	－	－	－
D-sorbitol	+	－	－
L-arabinose	+	+	－

+, positive; −, negative.

*Note*: Stains: *M. barrassiae* CIP108545^T^, *M. moriokaense* ATCC43059^T^. All data from the present study.

**Fig. 4.**
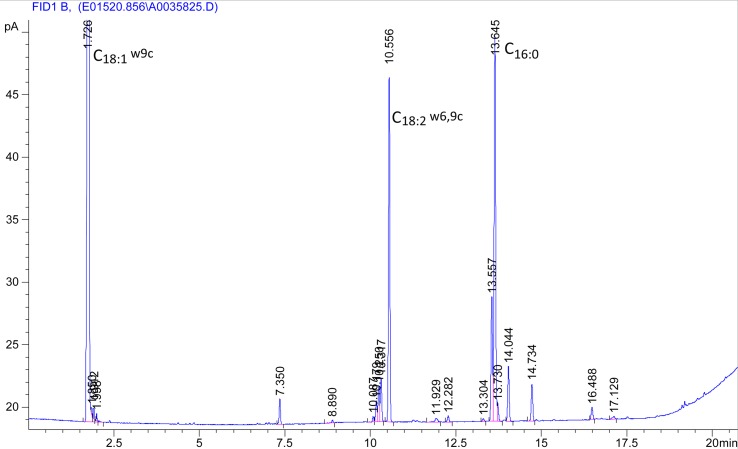
Fatty acid chromatogram of QGD101^T^.

The major fatty acids (>10% of the total fatty acids) disclosed by gas-liquid chromatography analysis detected in strain QGD101^T^ were C18:1*ω*9c (36.82%), C16:0 (29.9%) and C18:2*ω*6,9c (11.31%), for which the Sherlock Microbial Identification System (MIS) showed no match with QGD101^T^ (Fig. 4). And the DNA G + C content value of strain QGD101^T^ was 64.9% (65.25% ± 0.35%), which was consistent with the GC values sequenced from the genomic analysis.

### Drug resistance of strain QGD101^T^

The DST of strain QGD101^T^ showed that the MIC values of different drugs were: RFB (0.5 µg/ml), EMB (0.5 µg/ml), MOX (0.06 µg/ml), LVX (0.125 µg/ml), RIF (8 µg/ml), KAN (8 µg/ml) and AZI (128 µg/ml). According to the drug susceptibility criteria in the Susceptibility Test of Mycobacteria, Nocardia and Other Aerobic Actinomycetes [18], this result indicated that strain QGD101^T^ was sensitive to drugs RFB, EMB, MOX and LVX, but resistant to RIF, KAN and AZI.

## Discussion

In view of the development of environment and climate change, more mycobacteria are discovered and the membership list of genus *Mycobacterium* is ever expanding [19]. Therefore, discovering novel species of NTM and identification of clinical isolates are of great significance for the clinical practice. Especially, accurate differentiation of NTM from the *M. tuberculosis* complex is needed for effective patient management, because many NTM strains are resistant to the anti-mycobacterial agents [20]. This study suggested that the differential identification of mycobacteria is very significant to initiate personal medication in clinical practice, especially for the TB suspect.

In this study, a mycobacterium was isolated from a suspected TB patient. In order to accurately identify this bacteria, biochemical analysis, molecular sequence analysis, G + C content determination and cell wall fatty acid composition were performed. In most cases, 16S rRNA gene sequence can provide species-specific signature sequences useful for the identification of bacteria [21]. Further, the combination of multiple genes, 16S rRNA, *hsp65* and *rpoB* analysis is preferred [10 22 23]. The *rpoB* gene encodes the *β*-subunit of RNA polymerase containing conserved sequence regions flanking highly variable regions. It has been developed as a suitable tool for the accurate taxonomic identification of *Mycobacterium* [22]. The *hsp65* gene is a highly conserved housekeeping gene coding for a 65 kDa protein containing epitopes that are unique as well as epitopes that are common to various species of mycobacteria [24].

The *M. moriokaense* group was composed of *M. barrassiae* and *M. moriokaense*, sharing 99% identity in the 16S rRNA gene [25]. The strain QGD101^T^ shares 99% identity and 98% query cover with *M. barrassiae* CIP108545^T^ and *M. moriokaense* ATCC43059^T^ in the 16S rRNA gene. The phylogenetic tree of 16S rRNA suggested the close relationship of strain QGD101^T^ to *M. moriokaense* and *M. barrassiae*. By genomic analysis, QGD101^T^ showed 78.75% ANI values relative to the closest reference strain *M. moriokaense* ATCC43059T (NZ_MVIB01000100). These DNA relatedness and ANI results indicate that strain QGD101^T^ represents a novel genomic species that is distinct from its closest relatives.

The strain QGD101^T^ could grow on media containing either TCH or PNB indicating that QGD101^T^ is NTM. The differential characteristics between strain QGD101^T^ and the reference species of *Mycobacterium* were obvious, especially the ability to assimilate D-sorbitol, L-arabinose and the activity to *β*-galactosidase, *α*-glucosidase, suggesting that the strain QGD101^T^ represented a new species in the genus *Mycobacterium*.

To identify the drug resistance of this species, we chose MOX for patients. After 1 week of medication, the patient stopped coughing up blood, imaging examination showed that the lung shadow was weakened. After 2 months of treatment, the symptoms disappeared completely. This indicates that MOX can effectively inhibit the growth of the pathogen (QGD101^T^) in the patient, providing hope for the recovery of other patients infected with the strain.

In general, the physiological, biochemical characteristics and the genotype analysis demonstrated the clear differences between QGD101^T^ and the type strains of its closest phylogenetic neighbours. Therefore, the genome analysis indicated that strain QGD101^T^ represented a novel genomic species, and for which the name *Mycobacterium hubeiense* sp. nov. (type strain QGD101^T^ = CCTCCAA 2017003T = KCTC 39927T) was proposed.
